# Editorial: Experimental and Innovative Approaches to Multi-Target Treatment of Parkinson's and Alzheimer's Diseases

**DOI:** 10.3389/fnins.2022.910020

**Published:** 2022-05-16

**Authors:** Maria A. Tikhonova, Hung-Ming Chang, Sandeep Kumar Singh, Didier Vieau

**Affiliations:** ^1^Laboratory of the Experimental Models of Neurodegenerative Processes, Department of Experimental Neuroscience, Scientific Research Institute of Neurosciences and Medicine (SRINM), Novosibirsk, Russia; ^2^Department of Anatomy and Cell Biology, School of Medicine, College of Medicine, Taipei Medical University, Taipei, Taiwan; ^3^Indian Scientific Education and Technology Foundation, Lucknow, India; ^4^U1172 - LilNCog - Lille Neuroscience and Cognition, Alzheimer and Tauopathies, Université de Lille, Lille, France

**Keywords:** neurodegeneration, experimental therapy, neuromodulation techniques, animal models, multi-interventions, neuroinflammation, peripheral mechanisms

Alzheimer's disease (AD) and Parkinson's disease (PD) are incurable and the most common neurodegenerative disorders. Current methods for AD and PD treatment are mostly symptomatic, while a new effective pathogenesis-relevant therapy that would block the disease course and restore all the compromised functions is demanded. AD and PD pathogeneses are largely associated with the accumulation of neurotoxic protein aggregates in the brain, i.e. toxic forms of amyloid-beta (Aβ), alpha-synuclein, and tau protein (Selkoe and Hardy, 2016; Rocha et al., 2018). Hence, enhancing the pathological protein elimination has attracted increasing attention. In June 2021, the FDA approved the drug Aducanumab (brand name-Aduhelm) based on a monoclonal antibody against amyloid. However, the introduction of this drug into widespread clinical practice raises certain skepticism (Knopman and Perlmutter, 2021), mainly due to the uncertain therapeutic effect or clinical benefit. There is also a concern about targeting amyloid or alpha-synuclein directly since Aβ precursor protein and alpha-synuclein are both involved in normal physiological function (Dawkins and Small, 2014; Nellikka et al., 2021) and thus their content should not fall below the critical level. Failure of clinical trials of “Aβ-oriented” drugs may also be related to their use at the late stages of AD, whereas these agents could be effective when pathological aggregation of Aβ just begins preceding the initial signs of cognitive impairment in patients for at least 10–20 years (Frozza et al., 2018). Moreover, neurodegenerative disorders have a multifactorial etiology and involve various pathological processes in addition to neurotoxicity of protein aggregates, such as oxidative stress, neuro-inflammatory response, disturbed neurotrophic function and neurogenesis, synaptic and neurotransmission dysfunction, ion disbalance, etc. that often closely interact and overlap. Therefore, researchers develop multipurpose drug combinations (combination-drugs-multi-targets, CDMT) that do not cause adverse side effects (Sahoo et al., 2018). Multipurpose therapy aimed at various important pathogenetic hubs is a novel trend regarded as a promising strategy for AD and PD therapy.

Currently, a number of research teams work within the CDMT strategy. For example, a recent study from Japan reported on the prevention of neurodegenerative dementia by intranasal rifampicin and resveratrol combination in mice (Umeda et al., 2021). Series of studies revealed that antibiotic drug ceftriaxone within a strategy of drug reprofiling produces neuroprotective effects both in AD and PD models through suppressing the glutamate-induced excitotoxicity, modulating the expression of genes related to Aβ metabolism, enhancing neurogenesis, attenuating neuro-inflammatory response, and recovery of neuronal density (Ho et al., 2014; Weng et al., 2016; Tikhonova et al., 2017, 2018). Moreover, a combination of ceftriaxone with erythropoietin allowed reducing the dosage of ceftriaxone by 20 times with maintained efficiency in a PD model (Huang et al., 2015). Finally, the promising results of clinical trials of phase III in China of GV−971 for treating people with mild to moderate AD were reported recently (Xiao et al., 2021). GV-971 is an oligosaccharide derived from marine organisms that affects such pathogenetic mechanisms of AD development as inhibition of Aβ fibril formation, neuroinflammation, and recondition of dysbiosis of gut microbiota (Martins et al., 2020; Ettcheto et al., 2021).

The aim of this Research Topic was to provide an updated overview on the approach of multi-targeted therapy for AD and PD and related issues. Several research groups contributed interesting points of view on this subject and elucidated important current aspects of the problem.

The central theme of the Topic is masterly illustrated by Reich and Hölscher who provided an accurate review of acylated ghrelin as a multi-targeted therapy for AD and PD. The review illustrates the wide-ranging neuroprotective properties of the acylated form of the hormone ghrelin and discusses its potential to ameliorate pathologic changes occurring in AD and PD as well as complications of long-term treatment with the drug.

Tikhonova et al. contributed an original work in which multiple neuroprotective effects of antibiotic ceftriaxone against AD-like pathology are discussed, mainly focusing on mechanisms related to Aβ burden and neuro-inflammatory response.

Komleva et al. reviewed the pathological role of inflamm-aging, brain insulin resistance, and their cross-talk in aging and neurodegeneration. The review summarizes current knowledge on immunosenescence, inflamm-aging, and metainflammation and discusses potential mechanisms of calorie restriction as multi-purpose approach that may effectively break the vicious cycle of metainflammation, improve insulin resistance and slow the onset of neurodegeneration.

Studies on PD have mostly focused on processes and targets in the central nervous system. In clinical practice, neuromodulation techniques such as deep brain stimulation (DBS) are applied to control drug-resistant symptoms of PD. In a case report by Chang et al., a method of bilateral globus pallidus interna (GPi) combined with subthalamic nucleus (STN) variable frequency DBS (bSGC-DBS) implantation was introduced. The case of a young-onset PD patient with refractory dyskinesia explores multi-electrode and multi-target stimulation for the treatment of dystonia disorders.

On the other hand, a review by Ma et al. emphasizes the importance of the peripheral nervous system (PNS) in PD pathology. The paper discusses the use of pathological changes in PNS for clinical diagnosis of PD as well as the application of PNS targets for PD therapy, namely Schwann cell transplantation in the treatment of PD animal models is described. Wichit et al. contributed an original clinical work in which monoamine levels in peripheral body fluids were analyzed in association with clinical profiles in PD patients. The paper points to the involvement of several neurotransmission systems in PD pathology. An original research article by Seo and Yeo focuses on the alterations in muscle proteins that could impair muscle function and add to the bradykinesia and tremor in a pharmacological 1-methyl-4-phenyl-1,2,3,6-tetrahydropyridine (MPTP)-induced mouse model of PD.

Two groups contributed original research papers on the effects of electroacupuncture in mouse AD models and discussed underlying mechanisms. Xu et al. focused on the regulation of phospho-tau and glucose metabolism associated with the Akt/GSK3β signaling pathway while Xie et al. concentrated on M2 microglia polarization and glia anti-inflammation.

And last but not least, Ahanger et al. reviewed the role of monosodium glutamate in protein aggregation through a biophysical approach and discussed its potential impact on neurodegeneration.

Taken together, the papers collected in this Issue present the most recent knowledge and experimental evidence about the multi-target approach for therapy of neurodegenerative disorders and offer a new perspective and interesting hypotheses on this topic.

## Author Contributions

All authors listed have made a substantial, direct, and intellectual contribution to the work and approved it for publication.

## Funding

DV is supported by grants from Programmes d'Investissements d'Avenir LabEx (excellence laboratory) DISTALZ (Development of Innovative Strategies for a Transdisciplinary approach to Alzheimer's disease).

## Conflict of Interest

The authors declare that the research was conducted in the absence of any commercial or financial relationships that could be construed as a potential conflict of interest.

## Publisher's Note

All claims expressed in this article are solely those of the authors and do not necessarily represent those of their affiliated organizations, or those of the publisher, the editors and the reviewers. Any product that may be evaluated in this article, or claim that may be made by its manufacturer, is not guaranteed or endorsed by the publisher.
